# Post-Synthesis Modulation of the Physicochemical Properties of Green-Synthesized Iron Oxide Nanoparticles with Tween 80 to Enhance Their Antibacterial Activity and Biocompatibility

**DOI:** 10.3390/pharmaceutics17111371

**Published:** 2025-10-23

**Authors:** Marwa R. Bakkar, Alaa M. Ali, Gehad E. Elkhouly, Nermeen R. Raya, Terry W. Bilverstone, Nicholas P. Chatterton, Gary R. McLean, Yasmin Abo-Zeid

**Affiliations:** 1 Botany and Microbiology Department, Faculty of Science, Helwan University, Ain Helwan, Cairo 11795, Egypt; marwa_mahmoud01@science.helwan.edu.eg; 2 Department of Pathology, Faculty of Veterinary Medicine, Cairo University, Giza 12211, Egypt; alaamohamedali13@cu.edu.eg; 3 Department of Pharmaceutics and Industrial Pharmacy, Faculty of Pharmacy, Helwan University, Cairo 11795, Egypt; gehad.elkhouly@pharm.helwan.edu.eg (G.E.E.); nermeen.rayh@pharm.helwan.edu.eg (N.R.R.); 4 School of Life, Health and Chemical Sciences, The Open University, Walton Hall, Milton Keynes MK7 6AA, UK; terry.bilverstone@open.ac.uk (T.W.B.); nicholas.chatterton@open.ac.uk (N.P.C.); 5 School of Human Sciences, London Metropolitan University, 166-220 Holloway Road, London N7 8DB, UK; g.mclean@londonmet.ac.uk; 6 National Heart and Lung Institute, Imperial College London, Norfolk Place, London W2 1PG, UK

**Keywords:** green-synthesis, iron oxide nanoparticles, tween 80, antibacterial activity, in vivo acute irritation studies

## Abstract

**Background**: Iron oxide nanoparticles (IONPs) have broad-spectrum antimicrobial activity, with negligible potential for resistance development, excellent biocompatibility, and therefore, could be promising alternatives to conventional antimicrobials. However, their industrial-scale production relies on chemical synthesis that involves toxic reagents, imposing potential environmental hazards. In contrast, green synthesis offers an eco-friendly alternative, but our previous study found that green-synthesized IONPs (IONPs-G) exhibited a lower antibacterial activity and a higher cytotoxicity compared to chemically synthesized counterparts, likely due to nanoparticle aggregation. **Objectives**: To address this challenge, the current study presents a simple, effective, economic, scalable, and eco-friendly strategy to optimize the physicochemical properties of IONPs-G post-production without requiring extensive modifications to synthesis parameters. **Methods**: IONPs-G were dispersed in a solvent mixture containing Tween 80 (Tw80). Subsequently, in vitro antimicrobial and in vivo cytotoxicity studies on rabbits’ skin and eye were conducted. **Results**: The formed nanoparticles’ dispersion (IONPs-GTw80) had a particle size of 9.7 ± 2.1 nm, a polydispersity index of 0.111 ± 0.02, and a zeta potential of −11.4 ± 2.4 mV. MIC of IONPs-GTw80 values against *S. aureus* and *E. coli* were reduced by more than ten-fold compared to IONPs-G. MBC was twice MIC, confirming the bactericidal activity of IONPs-GTw80. In vivo studies of IONPs-GTw80 confirmed their biocompatibility with intact/abraded skin and eyes; this was further confirmed by histopathological and biochemical analyses. **Conclusions**: IONPs-GTw80 might be recommended as a disinfectant in healthcare settings or a topical antimicrobial agent for treatment of infected wounds. Nevertheless, further studies are required for their clinical translation.

## 1. Introduction

Antimicrobial resistance is a threat to global health [1,2,3] and economy [4,5]. This raises the crucial need for strong and firm infection control protocols in addition to effective guidance in healthcare settings [3,4,6,7], with the urge for developing alternative, effective, eco-friendly, and safe antimicrobial agents to combat multidrug-resistant bacteria.

Iron oxide nanoparticles (IONPs) are reported for their broad-spectrum antimicrobial activities, with minimal potential of microbial resistance development [8,9,10,11,12,13] and excellent compatibility with biological systems [14]. Their industrial manufacture is conducted by the chemical co-precipitation method, which involves hazardous and toxic reagents that pose environmental risks [15]. Consequently, eco-friendly green synthesis approaches for production of metal-based nanoparticles, including IONPs, are generally more favored over chemical synthesis [13,16,17].

Green synthesis approaches can involve the biological synthesis of metal-based nanoparticles using bacteria, fungi, or application of plant extracts to bio-reduce metal ions into nanoparticles [18]. Compared with microbial synthesis, applying plant extracts for green synthesis offers a more economical synthesis method [19,20,21,22]. Contrary to microbial synthesis systems that require a pollution-free source of insulation to assure safety and neat production [19]. Collectively, plant extracts might offer a more favorable route for green synthesis of metal-based nanomaterials at an industrial scale. However, several limitations are reported for green synthesis using plant extracts, such as low yield of nanoparticles and the incomplete understanding of the potential toxic effects associated with plant components [23]. In addition, numerous factors such as plant type, volume ratio of the extract, precursor concentration, stirring rate, incubation time, pH, and temperature of the reaction mixture are reported to significantly influence the key physicochemical properties of plant-derived metal-based nanomaterials, including size, polydispersity, shape, and surface charges [23,24,25]. Therefore, optimizing the key physicochemical properties of plant-derived metal-based nanomaterials to enhance their antibacterial activity requires extensive investigation of these factors. As a result, plant extract-based methods are often labor-intensive, time-consuming, and costly, thus limiting their feasibility for industrial applications [26,27,28,29]. Consequently, finding other strategies to modulate or optimize the physicochemical properties of green-synthesized nanoparticles without the need for exhaustive investigation of these factors could facilitate their industrial applicability.

In our previous study, IONPs were synthesized by chemical and green methods [30]. Our results showed that chemically synthesized IONPs had greater antibacterial activity and biocompatibility when compared to green-synthesized IONPs (IONPs-G). The low antibacterial activity and high cytotoxicity of IONPs-G were correlated to their strong tendency to aggregate, as revealed by their particle size and polydispersity index (PDI). Therefore, optimizing the physicochemical properties of IONPs-G with the objective of limiting their aggregation tendency might enhance their antibacterial efficacy and biocompatibility.

To the best of the authors’ knowledge, no previous studies have investigated the post-synthesis modulation of the key physicochemical properties of plant-derived metal-based nanoparticles. The present study aims to introduce a simple, effective, scalable, economical, and eco-friendly strategy to modulate the key physicochemical characteristics of green-synthesized IONPs after their synthesis with the objective of reducing their particle size and polydispersity, thereby avoiding the need for the extensive and time-consuming investigations of multiple influencing factors [23,24,25]. This involved synthesis of IONPs-G following our previously published protocol [30] and confirming their successful synthesis by XRD, FTIR, and XPS analysis. Subsequently, IONPs-G were dispersed in a selective solvent mixture containing Tween 80 (Tw80). Thereafter, the key physicochemical properties of the formed nanoparticles’ dispersion (IONPs-GTw80), including particle size, PDI, zeta potential, as well as morphological examination by TEM and HRTEM, were assessed.

Tw80 is a well-known non-ionic surfactant extensively used in the food and cosmetics industries due to its high biocompatibility and biodegradability, making it an eco-friendly and safe material [31,32,33]. In addition, Tw80 is well known to stabilize and minimize aggregation of metallic NPs, for example [34], and was therefore employed in the present study to stabilize IONPs-G and prevent their aggregation. After characterization of IONPs-GTw80, they were screened for their antibacterial activity against *Staphylococcus aureus* (*S. aureus*) and *Escherichia coli* (*E. coli*) to determine any improvements in efficacy. Furthermore, their in vivo biocompatibility was evaluated through acute irritation studies conducted on the skin and eyes of rabbits.

## 2. Materials and Methods

### 2.1. Materials

Ferric chloride anhydrous (purity 98%) was purchased from Acros Organics and Oxford reagents, respectively. Quercus Infectoria Galls (QIG) powder was obtained from the Egyptian market and it was authenticated by Professor Dr. Suzanne Michail, Professor of Pharmacognosy, Faculty of Pharmacy, Helwan University, Cairo, Egypt. All microbiological media used in the current study were purchased from Hi-Media, Thane, India. Peptone and sodium chloride were purchased from Oxoid, Basingstoke, UK. Dimethyl sulfoxide (DMSO) was obtained from Honeywell™, Charlotte, NC, USA. 3-(4,5-Dimethylthiazol-2-yl)-2,5-diphenyltetrazolium Bromide (MTT) was obtained from Serva, Heidelberg, Germany, and phosphate buffer saline (PBS) tablets were obtained from Merck, Darmstadt, Germany. All other chemicals and reagents employed were of analytical grade. Pathogenic bacteria were collected from a clinical setting in Cairo, Egypt: *S. aureus* and *E. coli*. These were identified as multidrug-resistant bacteria (MDR) and extensively drug-resistant (XDR), respectively (Table S5, Supplementary File).

### 2.2. Methodology

#### 2.2.1. Synthesis and Characterization of IONPs-G

IONPs-G was synthesized using the extract of the plant powder of the outgrowth formed by the deposition of eggs from Cynips Gallae tinctoriae, a member of the Cynipidae family, on the young twigs of Quercus infectoria (QI), a member of the Fagaceae family, following our previously published protocol [30]. Briefly, 18 mL of ferric chloride solution (2.5 mM, Mwt 162.21) in double-distilled water (DDW) was homogenized with 30 mL of ethanol (10% *v*/*v*) using a Daihan Homogenizer (ZS version, Wonju-si, Korea) at 750 rpm for 6 min. Subsequently, 2 mL of freshly prepared plant extract was added dropwise under continuous homogenization at 750 rpm for 7 min. The appearance of a black color indicated the successful synthesis of IONPs-G. The reaction mixture was left to stir overnight to allow ethanol evaporation, followed by drying at 150 °C for 5 h in an oven (Rumo Model G25, Cairo, Egypt) to obtain the black powder of IONPs-G. The synthesized IONPs-G were characterized using XRD, FTIR, and XPS to confirm their successful synthesis in advance of proceeding to further experimental studies. The major metabolites of the aqueous extract of Quercus infectoria galls (QIGs) were successfully identified using high-performance liquid chromatography–mass spectrometry (LC-MS/MS) and the obtained data (Table S1 in Supplementary File) was identical to what was reported in our previous publication [30].

#### 2.2.2. Preparation and Characterization of IONPs-GTw80 Colloidal Dispersion

IONPs-G powder (10 mg) was dispersed in 1 mL of absolute ethanol, followed by stirring on a plate stirrer (DAIHAN Scientific, Wonju-si, Republic of Korea) at a moderate speed for 15 min to ensure effective dispersion of the nanoparticles. Subsequently, 18 mL of 10% ethanol was added, followed by 10 mL of Tw80. Then, another 21 mL of 10% ethanol was added, and the mixture was homogenized (Daihan Homogenizer, ZS version, Korea) at 750 rpm for 14 min. The resulting IONPs-GTw80 colloidal dispersion was characterized using dynamic light scattering (DLS) for particle size, PDI, and zeta potential using a Malvern Zeta-sizer Nano ZS (Malvern Instruments Ltd., Malvern, Worcestershire, UK). The sample was diluted in DDW to achieve a count rate of 50–300 Kcps, with measurements taken at 25 ± 0.1 °C and a measurement angle of 173°. The morphology of IONPs-GTw80 was observed using TEM (H-700, Hitachi Ltd., Tokyo, Japan), following our previously published protocol [2,30,35,36]. High-resolution transmission electron microscopy (HRTEM) was conducted for IONPs-GTw80 as follows: nanoparticles were dispersed in DDW and a 3–5 μL droplet of the suspension was deposited onto a glow-discharged, carbon-supported transmission electron microscopy (TEM) grid (EM Resolutions, Keele, UK). The grid was left to air-dry overnight. High-resolution TEM (HR-TEM) images were captured using a JEOL JEM-2100 microscope (JEOL, Tokyo, Japan) operating at 200 kV. Images were acquired at magnifications between 200,000× and 400,000× using an 11-megapixel Gatan Orius in-line digital camera (Ametek GB Limited, Leicester, UK).

The stability of IONPs-GTw80 versus IONPs-G in PBS (10 mM, pH 7.4) was tracked over 24 h by measuring particle size, PDI, and zeta potential. To conduct the stability test, 200 µL of IONPs-G/IONPS-GTw80 dispersed in DDW was mixed with PBS (10 mM, pH 7.4, 800 µL), followed by measuring the particle size, PDI, and zeta potential at 0, 1, 3, 6, and 24 h. A blank sample for IONPs-GTw80 was prepared using the same procedure, but without the addition of IONPs-G, to be used as a control in microbiological and in vivo safety studies.

#### 2.2.3. Microbiological Studies

##### Microdilution Assay

The antibacterial activity of IONPs-GTw80 was screened following a microdilution test against *S. aureus* and *E. coli* according to our previously published protocol [7,30,36,37]. According to Clinical and Laboratory Standards Institute guidelines, Muller-Hinton broth (MHB) was used to determine minimum inhibitory concentration (MIC) and minimum bactericidal concentration (MBC) [38]. In brief, a fresh inoculum of each tested bacterial pathogen (5 log CFU/mL) was prepared. In a 96-well microplate, the colloidal dispersion of IONPs-GTw80 (100 µL) was added into each well, followed by a two-fold serial dilution using sterile MHB. Subsequently, bacterial inoculum (10 µL) was then inoculated per well. Inoculated plates were aerobically incubated overnight. At the end of the incubation, plates were examined for MIC and MBC [39] by inoculating each well with MTT solution (10 µL, 5 mg/mL, PBS) followed by further incubation at 37 °C for 2 h. To solubilize the formed crystals of formazan, DMSO (100 µL/well) was added and plates were left for 2 h at room temperature. Absorbance was measured at 570 nm using a Microplate Reader Spectrophotometer (Bio-Tek MQX200 uQuant; BioTek Instruments, Winooski, VT, USA). MIC was described as the lowest concentration of IONPs-GTw80 required to inhibit bacterial growth by assessing turbidity visually, while MBC was identified as the lowest concentration of IONPs-GTw80 required to kill bacteria, as determined by a lack of bacterial colonies when plated onto Muller–Hinton agar (MHA) plates.

To ascertain the reliability of the microbiological test, the following set of controls was evaluated for their antibacterial activity: (1) the plant extract used for synthesis of IONPs-GTw80, and (2) the blank sample for IONPs-GTw80 prepared as described in Section 2.2.2. Moreover, a set of controls for each bacterium was prepared: (3) sterile MHB culture media free of NPs or bacteria “negative control 1”, (4) sterile MHB culture medium containing serial dilution of NPs “negative control 2” only, and (5) MHB culture media inoculated only with bacteria “positive control”.

##### Time–Kill Curve Study of IONPs-GTw80

An experiment of time–kill curve was conducted according to our previously published protocol [7,37] to determine the effective duration of the antibacterial activity of IONPs-GTw80 colloidal dispersion. In brief, a fresh bacterial culture of *S. aureus* and *E. coli* containing 5 log CFU/mL was exposed to MBC of IONPs-GTw80 colloidal dispersion. At predetermined time intervals, an aliquot of the inoculum was taken and serially diluted with a solution of 0.9% NaCl and 1% peptone. Subsequently, 100 µL of each dilution was then spread over the surface of dried TSA plates, followed by incubation for 18–24 h at 37 °C. Afterwards, plates were observed for bacterial growth and colonies were counted and compared to control plates. The growth inhibition percentage was calculated relative to the control (a bacterial culture without IONPs-GTw80). For reliability results, two independent experiments were performed, with triplicate for each concentration.

##### TEM Study

Following our previously published protocol [7,37,40], overnight fresh cultures of *S. aureus* and *E. coli* were individually incubated with IONPs-GTw80 colloidal dispersion at its MBC for 2 h. Bacterial culture was first centrifuged and the collected pellet containing bacterial cells was washed twice with PBS (1.5 mL, pH 7.2), followed by a fixation step using glutaraldehyde in PBS (2% *v*/*v*). A post-fixation step was conducted at room temperature for 1 h using OsO_4_ (1% *w*/*v*) in PBS (5 mmol/L). Following three successive washes with PBS, the samples were dehydrated through an ethanol series and subsequently embedded in epoxy resin. Sections with thickness of about 500–1000 µm were prepared using a microtome (Leica ultra-cut microtome). These sections were stained with toluidine blue and investigated with a 1× magnification lens, where images were captured by a Leica ICC50 HD camera ( Leica Microsystems, Wetzlar, Germany). Sections of around 75–90 µm thickness were then prepared and stained twice with saturated uranyl acetate and lead citrate. Stained sections were then examined using TEM (JEM-1400 TEM, JEOL-Hitachi, Tokyo, Japan) at different magnifications. Images were captured using an AMT CCD Optronics camera (Advanced Microscopy Techniques Corp., Danvers, MA, USA) (1632 × 1632-pixel format), with a 1394 FireWire board for acquisition.

#### 2.2.4. Acute Irritation Study

##### Animals

New Zealand white rabbits (CLAVCAP-VACSER, Cairo, Egypt), with weights ranging from 2.5 to 3 kg, were used in the current study to track the incidence of any irritation that might occur due to the topical application of IONPs-GTw80 colloidal dispersion on the eyes and skin (intact/abraded). The rabbits lived in conventional housing circumstances, with a controlled 12-hour light/dark cycle, a temperature of 25 ± 2 °C, and a relative humidity of 50 ± 20%. Animals were given unrestricted access to a balanced commercial pelleted meal that contained at least 5% fiber, 20% protein, 3.5% fat, and 6.5% vitamins and ash. They also had unlimited access to fresh water. Before the trial began, all animals were acclimatized to the laboratory setting for one week under stringent hygienic conditions. The Ethics Committee of the Faculty of Veterinary Medicine at Cairo University (Vet CU20092022459) examined and approved the experimental protocols. Throughout the investigation, every attempt was made to minimize animal discomfort or distress.

##### Skin Irritation Test

Skin irritation test was performed following our previously published protocol [2,30]. Briefly, one day prior to the experiment, each of the 24 rabbits had a 1.5 × 1.5 cm^2^ section of skin marked and shaved with an electric razor. Twelve rabbits each were randomly assigned to one of two primary groups: those with intact skin and those with abraded skin. Each main group was further divided into three sub-groups, each comprising 4 rabbits. Subgroup I: untreated control group; Subgroup II: rabbits received 0.5 mL of the blank sample for IONPs-GTw80, prepared as described in Section 2.2.2; Subgroup III: rabbits were treated with 0.5 mL of IONPs-GTw80 colloidal dispersion at a concentration of 200 µg/mL, corresponding to its MBC. All shaved skin portions were wrapped with sterile gauze of the same size and held in place with tape that did not irritate the skin. We looked for symptoms of irritation, like erythema and edema, on both intact and abraded skin at 24, 48, and 72 h, using the Magnusson and Kligman scale [41]. All animals were photographed after 72 h of treatment. Subsequently, all animals were euthanized using cervical dislocation, and the skin was excised for histological evaluation [2,41].

##### Eye Irritation Test

The Draize modified test was conducted in accordance with our previously published protocol [2,30] to assess the ocular irritation that might occur due to application of IONPs-GTw80 colloidal dispersion (200 µg/mL) and its blank sample in 10 white New Zealand rabbits, where five rabbits were used for each sample [42]. The cornea was exposed to the sample by instilling 50 µL of each sample into the lower cul-de-sac of the rabbits’ eyes. The left eye was administered the sample five times at 5-minute intervals, while the right eye served as a negative control (untreated). We delicately shut the animal’s eyes for approximately 10 s to avert sample loss. The rabbits’ eyes were meticulously observed for possible ocular responses, including erythema, conjunctival chemosis, discharge, and lesions of the cornea and iris. Observations were conducted at consistent intervals of 24 and 72 h, utilizing a histological grading system to evaluate ocular discomfort [43]. Grades ranging from 0 to 3 were assigned based on the presence and severity of symptoms such as inflammation, redness, and tearing, with higher scores indicating more pronounced irritation [44]. Images were captured for all groups 72 h following the commencement of treatment.

##### Histopathological Evaluations

The animals were killed after 72 h of treatment using ether anesthesia. The removed dorsal skin and eyes were delicately cleaned with ice-cold PBS. Samples were subsequently fixed in 10% neutral buffered formalin (HT501128, Sigma-Aldrich, Burlington, MA, USA; comprising about 10% formaldehyde in PBS, pH 6.9–7.1). Subsequent to fixation, tissues underwent processing through graded ethanol for dehydration, were cleaned in xylene (A5597, Sigma-Aldrich), and were embedded in paraffin. Paraffin-embedded blocks were sliced to a thickness of 4–5 µm and stained with hematoxylin and eosin (H&E) using the protocol established by Bancroft and Gamble (2008) [45].

Histopathological findings in the skin were evaluated and rated semi-quantitatively according to [45] (Bancroft and Gamble 2008), with the following modifications: 0 indicates no anomaly, 1 signifies little abnormality, 2 denotes mild abnormality, and 3 represents substantial abnormality. The evaluation was conducted on seven observations: hypertrophy, hyperkeratosis, parakeratosis, erosion, inflammatory cell infiltration, extracellular edema, and ulceration. All specimens were subjected to histological investigation at the Faculty of Veterinary Medicine, Cairo University.

##### Biochemical Examination

Alanine aminotransferase (ALT) and alkaline transferases (ALKs) (Wuhan, China), markers of liver function, were measured in serum samples from rabbits in all groups using EIAab^®^ kits (Wuhan, China) to assess liver function.

##### Hematological Examination

An ABC animal blood count apparatus (Vet 907 AB 6012) was applied to get a complete blood count (CBC).

#### 2.2.5. Statistical Analysis

Analysis of variance (ANOVA), followed by the Tukey post hoc test for comparisons between multiple groups, was conducted to statistically analyze data obtained in the current study. GraphPad Prism 9.0 software at a confidence level of 95% was used to carry out the analysis.

## 3. Results

### 3.1. Green Synthesis and Characterization of IONPs

The successful synthesis of the new batch of IONPs-G was confirmed by XRD, FTIR, and XPS (Figures S1–S3 in Supplementary File) and it was consistent with our previous publication [30]. Afterwards, IONPs-G was dispersed in a selected solvent mixture containing Tw80, and its characterization via XRD, FTIR, and XPS was conducted (Figures S1–S3, in Supplementary File). Subsequently, the key physicochemical properties of the formed IONPs-GTw80 were determined and presented in Table 1. The particle size was presented as an average particle size (D nm) ± standard deviation (SD) and was 9.7 ± 2.1 nm, PDI was 0.111, and the zeta potential value was expressed as average zeta potential (mV) ± SD and was −11.4 ± 2.4 mV. Despite the low negativity of the zeta potential value recorded, the PDI value indicated a homogenous distribution of IONPs-GTw80 in DDW with a lower tendency of aggregation [46] compared to that reported for IONPs-G in our previous publication, where the PDI value was ≥0.5 [30].

UV–visible spectrophotometric analysis of IONPs-GTw80 and IONPs-G (Figure S4 in Supplementary File) revealed their absorption peaks to be 294.5 and 325 nm, respectively. The difference observed for the wavelength of absorption peaks could be linked to their particle size [47].

**Figure 1:**
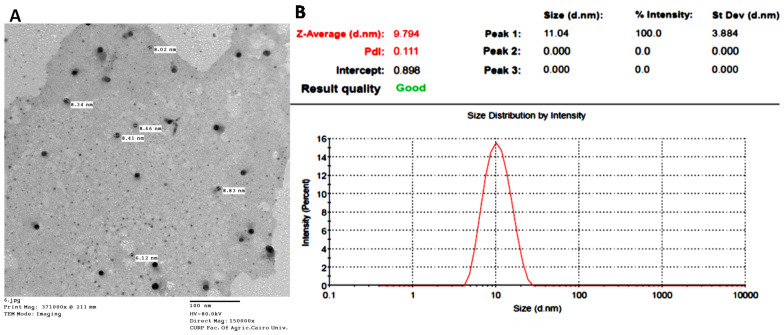
Characterization of IONPs-GTw80; transmission electron microscopy (TEM) image, scale bar 100 nm (**A**); dynamic light scattering (DLS) particle size and polydispersity index (PDI) (**B**).

TEM image (Figure 1A) revealed particles with a spherical morphology; however, the particle size ranged from 6.12 to 8.83 nm, and was slightly lower than the particle size recorded by the Malvern instrument (Figure 1B). HRTEM image of IONPs-GTw80 (Figure S1C) further demonstrated the spherical morphology of NPs.

The stability study conducted for IONPs-G versus IONPs-GTw80 in PBS over 24 h (Table S2 and Figure S5 in the Supplementary File) revealed that IONPs-G had a larger particle size than IONPs-GTw80 with a higher tendency to aggregate. The particle size of IONPs-G and IONPs-GTw80 ranged from 620.9 ± 36.55 to 887.97 ± 87.88 nm, and 12.037 ± 0.03 to 24.453 ± 0.78 nm, respectively. Zeta potential values recorded for IONPs-G and IONPs-GTw80 dispersed in PBS and ranged from −31.23 ± 1.89 to −34.00 ± 1.05 mV, and −18.20 ± 1.31 to −26.80 ± 1.13 mV, respectively. Although the zeta potential value recorded for IONPs-GTw80 was lower than that recorded for IONPs-G, the measured PDI values for IONPs-GTw80 indicated their stability and their lower tendency to agglomerate compared to IONPs-G. PDI value recorded for IONPs-G dispersed in PBS was ≥0.5, indicating a polydisperse sample [46]. In contrast, PDI measured for IONPs-GTw80 was ≤0.37, indicating a sample with a narrow size distribution with less tendency of particle aggregation [46].

### 3.2. Antibacterial Activity

#### 3.2.1. Determination of MIC and MBC

MIC and MBC values of IONPs-GTw80 were determined as described in Section Microdilution Assay and presented in Table 2.

Neither the blank sample for IONPs-GTw80 prepared as described in Section 2.2.2, nor the plant extract showed any antibacterial activity, and this indicated that the antimicrobial activity is solely due to IONPs-GTw80. MIC and MBC data revealed that IONPs-GTw80 exhibited the same antibacterial activity against both Gram-positive and Gram-negative bacteria and this is consistent with our previously obtained results for IONPs-G [30]. However, when comparing the results of this study to our previous study [30], IONPs-GTw80 appears to have a more efficient antibacterial activity compared to IONPs-G, as revealed from MIC and MBC values recorded for IONPs-GTw80 that were more than ten-fold and five-fold lower than those recorded for IONPs-G (1000 µg/mL), respectively.

#### 3.2.2. Time–Kill Curve Study

**Figure 2:**
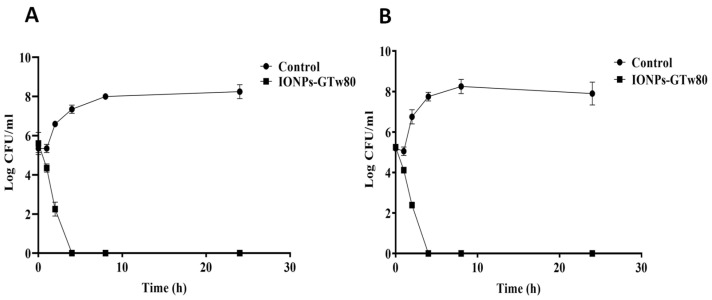
Time–kill curve for IONPs-GTw80 against *S. aureus* (**A**), and *E. coli* (**B**). Two independent experiments were conducted with triplicate for each one. Error bars represent the standard deviation at each point.

The time–kill curves for IONPs-GTw80 against *S. aureus* and *E. coli* are presented in Figure 2. As presented, cell density for both types of bacteria was reduced by half after 2 h when incubated with MBC of IONPs-GTw80. Moreover, a complete eradication of both bacteria occurred after 4 h of incubation with IONPs-GTw80, indicating that IONPs-GTw80 has an efficient bactericidal effect.

#### 3.2.3. Transmission Electron Microscopy (TEM)

**Figure 3:**
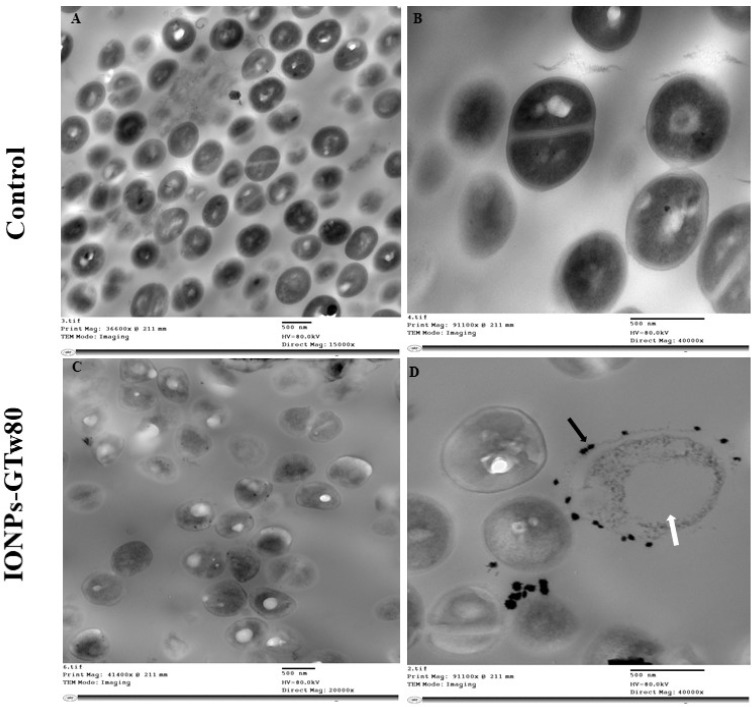
TEM images showing the resultant changes in *S. aureus* structures after being treated with IONPs-GTw80 at MBC. Images (**A**,**B**) show normal bacterial cell without any cell structure abnormality, with intact cell wall, normal cell division and intracellular components. Images (**C**,**D**) show structural changes observed after treatment with IONPs-GTw80. Image (**C**) provides an overview of abnormal cell morphology with the presence of IONPs-GTw80 in the field. Image (**D**) shows almost destructed cell with large vacuole (white arrow) and the presence of IONPs-GTw80 on cell surface (black arrow).

In order to explore the mechanism of antibacterial activity for IONPs-GTw80 against *S. aureus* and *E. coli*, TEM was conducted to compare the ultrastructural changes of treated bacterial cells with those of control untreated ones. TEM images (Figure 3A,B) showed untreated *S. aureus* with normal shape and structure, where the cell wall appeared intact with a typical cell division. Upon treating *S. aureus* with IONPs-GTw80, several structural deformations were observed (Figure 3C). This involved distorted cell morphology, with IONPs-GTw80 distributed on the cell wall of bacterial cells (Figure 3D, black arrow), and formation of a large cavity resembling a vacuole (Figure 3D, white arrow).

**Figure 4:**
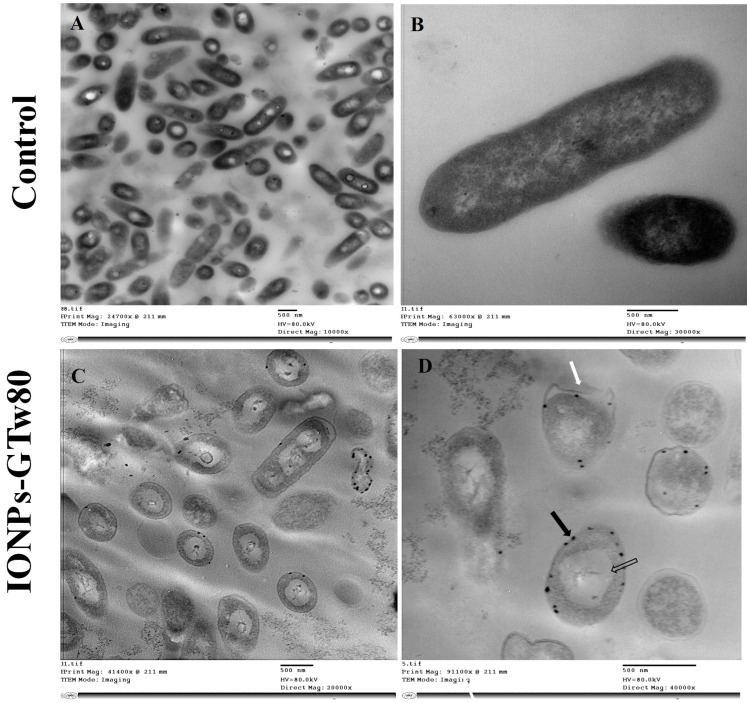
TEM images describing the resultant changes in *E. coli* structure after being treated with IONPs-GTw80 at MBC. Images (**A**,**B**) show fields of normal bacterial cells with no observed structural changes with intact cell wall, and normal intracellular components. Images (**C**,**D**) show abnormality observed after treatment with IONPs-GTw80. Image (**C**) shows an overview of abnormal cell structures and distribution of IONPs-GTw80 in the field. Image (**D**) shows cells with detached cell wall margin (white arrow) with nanoparticles distributed on treated bacterial surface (black arrow) and large vacuole (hollow arrow).

*E. coli* treated with IONPs-GTw80 also showed cell abnormality when compared to untreated bacterial cells. TEM images revealed that untreated *E. coli* cells have a normal cell shape without any observed abnormalities in cell structure (Figure 4A,B). *E. coli* treated with IONPs-GTw80 revealed large, massive deformations of bacterial internal structure (Figure 4C). Again, IONPs-GTw80 was distributed on the surface of cell wall of treated bacteria (Figure 4D, black arrow). In addition, a detached cell wall (Figure 4D, white arrow) and formation of a large vacuole (Figure 4D, hollow arrow) were also observed.

### 3.3. Irritation Test

#### 3.3.1. Skin Irritation Test

**Figure 5:**
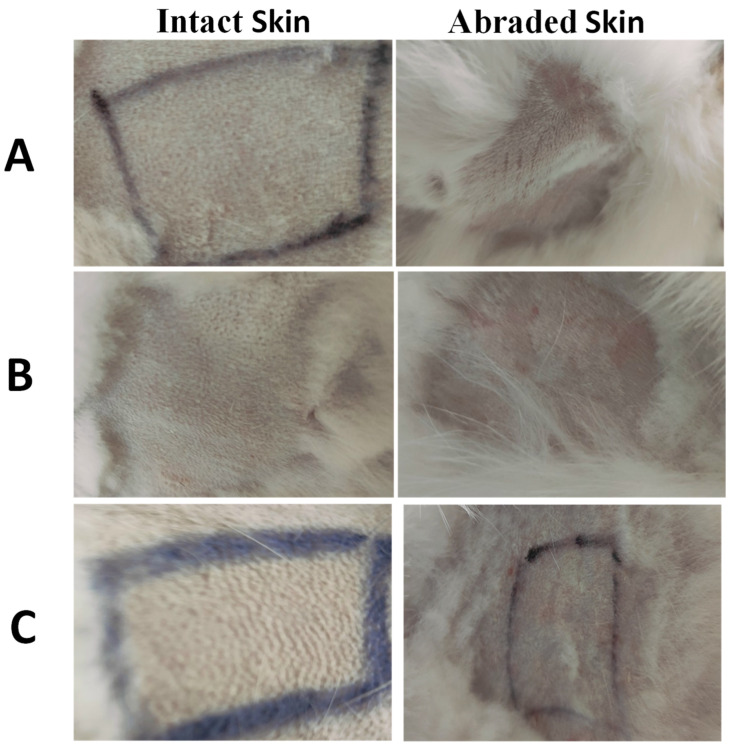
Images of representative rabbits from the skin irritation test for an untreated animal (negative control) (**A**), animal treated with IONPs-GTw80 (200 µg/mL) (**B**), and animal treated with blank solution (**C**). A non-irritating marker was used to draw black lines. In comparison to untreated animal, no differences in score were observed after 72 h for sites treated with IONPs-GTw80 and its blank solution for both abraded and intact skin.

As presented in Figure 5 and Table S3 in the Supplementary File, for all tested samples, both abraded and intact skin treated with IONPs-GTw80 and its corresponding blank sample revealed no differences when compared to the untreated animals across all evaluated parameters, including erythema, eschar, and edema. The control areas on the abraded skin exhibited erythema in contrast to the intact skin, potentially linked to the needle abrasion. Nevertheless, the erythema diminished, and the dermis reverted to its baseline condition within 30 to 45 min.

#### 3.3.2. Eye Irritation Test

**Figure 6:**
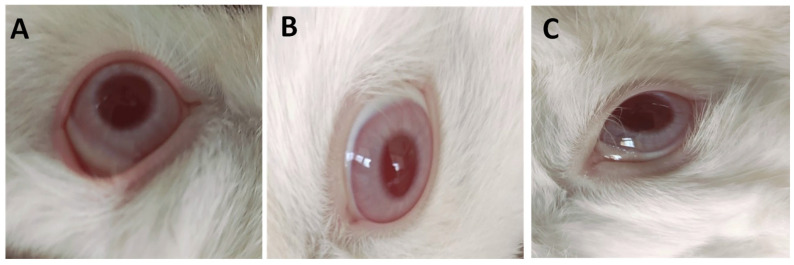
Images of representative rabbits from eye irritation test for eyes of an untreated animal (negative control) (**A**), eyes treated with IONPs-GTw80 (200 µg/mL) (**B**), eyes treated with blank solution (**C**). The cornea, iris, and conjunctiva were observed after 72 h of treatment. Cornea, iris, and conjunctiva of groups treated with IONPs-GTw80 and its blank solution showed no differences compared to untreated eyes.

Figure 6 and Table S4 in the Supplementary File revealed that untreated (negative control) eyes and those treated with IONPs-GTw80 (200 µg/mL), and its blank solution, did not induce any irritation or abnormalities. The images in Figure 6 reveal no differences between untreated and treated eyes, with no sign of abnormalities in the cornea, iris, and conjunctiva in both treated and untreated eyes. This indicates the compatibility of IONPs-GTw80 and its blank solution with eye tissues. It is important to note that pupil sizes varied among the animals, which was attributed to the different light levels while taking photos.

#### 3.3.3. Histopathological Evaluations

**Figure 7:**
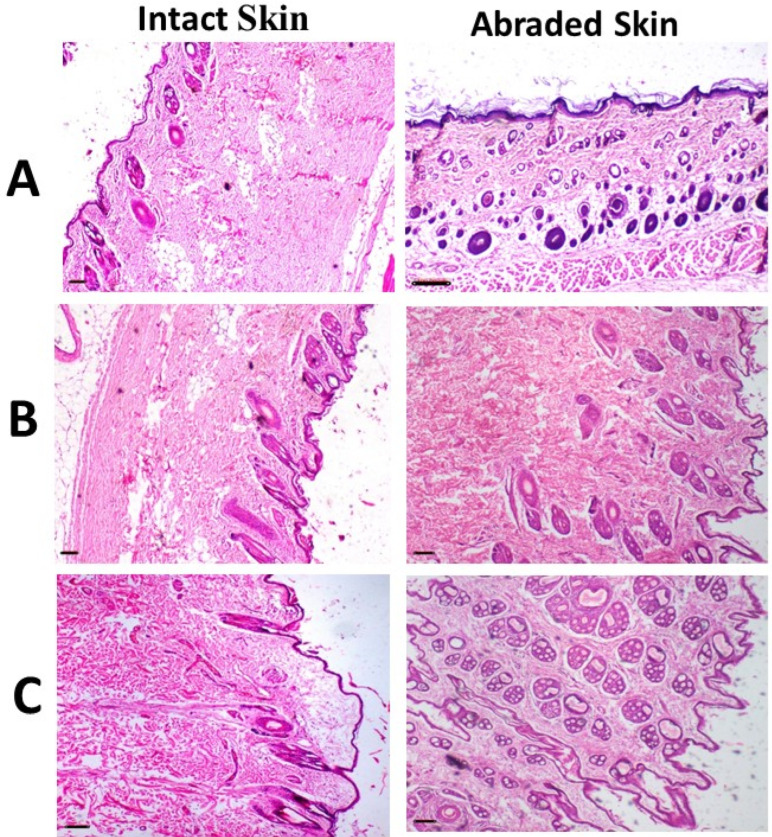
Images of representative rabbits of H and E stained photomicrographs of skin for an untreated animal (negative control) (**A**), animal treated with IONPs-GTw80 (200 µg/mL) (**B**), and animal treated with blank solution (**C**). A normal histological tissue identical to untreated animal was observed after 72 h of treatment for all tested samples. Skin layers for tested samples were normal and similar to those of untreated animal with no sign of erosion, ulcers, necrotic cells, or inflammatory cells. All photomicrographs were taken at 4× objective magnification.

The histopathological results of the skin irritation test for both intact and abraded skin are presented in Figure 7. The data indicated that animals administered IONPs-GTw80 (200 µg/mL) and the blank solution exhibited skin with typical histology, comparable to that of untreated animals. The skin layers of both intact and abraded skin had normal morphology, devoid of erosion, ulcers, necrotic cells, or inflammatory cells.

**Figure 8:**
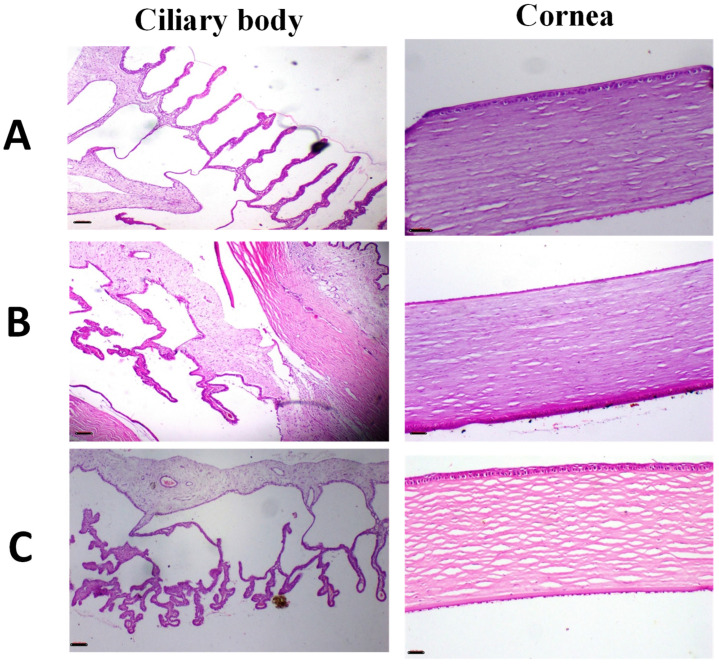
Images of representative rabbit of H and E stained photomicrographs for eyes of an untreated animal (negative control) (**A**), eyes treated with IONPs-GTw80 (200 µg/mL) (**B**), and eyes treated with blank solution (**C**). A normal histological eye tissue identical to untreated animal was observed after 72 h of treatment for all tested samples. Cornea, fibrous connective tissue showed no signs of inflammation, erosion, ulcers, or necrobiotic changes. Ciliary body revealed no cutting in the filament of ciliary body with no edema. All photomicrographs were taken at 4× objective magnification.

Histopathological evaluations of the eye depicted in Figure 8 revealed that the cornea and fibrous connective tissue in both untreated eyes and those treated with IONPs-GTw80 and the blank solution appeared normal, exhibiting no indications of inflammation, erosion, ulcers, or necrobiotic alterations. The ciliary body had normal characteristics, with no disruption of the ciliary body filaments or signs of edema. The results indicate that IONPs-GTw80 and its control solution are compatible with ocular tissues.

#### 3.3.4. Biochemical Analysis

Liver function biomarkers were measured in the serum samples of untreated rabbits and those treated topically with IONPs-GTw80 (200 µg/mL) and its blank solution. The results, presented in Table 3, showed that the levels of alanine aminotransferase (ALT) and alkaline transferase (ALK) recorded in animals treated with IONPs-GTw80 and the blank solution showed non-significant (*p* > 0.05) differences when compared to untreated animals. This suggests that the topical application of IONPs-GTw80 and its blank solution did not induce any liver toxicity.

#### 3.3.5. Hematological Examination

The hematological evaluation for animals treated topically with IONPs-GTw80 (200 µg/mL), the blank solution, and untreated animals is presented in Table 4. The mean values of the erythrogram for the treated animals did not significantly differ (*p* > 0.05) from those of the untreated animals

## 4. Discussion

In a previous study [30], our group synthesized and characterized IONPs-G, and demonstrated their antibacterial activity against *S. aureus* and *E. coli*; however, MIC and MBC values recorded for both bacteria were >1000 µg/mL, which were much higher than those recorded for IONPs produced by chemical synthesis [IONPs coated with cetyltrimethylammonium bromide (CTAB)], where MIC and MBC recorded against *S. aureus* were 31.25 and 62.5 µg/mL, while those recorded against *E. coli* were 62.5 and 125 µg/mL, respectively. The high values of MIC and MBC of IONPs-G were attributed to their high tendency to aggregate. The aggregation of IONPs-G was also linked to its observed adverse effects when applied to the rabbits’ abraded skin, where moderately edematous, red, and inflamed skin was observed. Furthermore, edematous conjunctiva and cornea erosion were also observed after treatment of rabbits’ eyes [30].

The objective of the current study was to prepare less aggregated green-synthesized IONPs to enhance their antibacterial activity and minimize their adverse effects in a facile manner, which could facilitate their industrial applicability. This involved synthesis of a new batch of IONPs-G, and their successful synthesis was confirmed by XRD, FTIR, and XPS analyses (Figures S1–S3 in Supplementary File). The drying of IONPs-G was conducted at 150 °C, as at higher temperatures, a more aggregated powder that is difficult to re-disperse was obtained. This is consistent with the literature [48], where drying at higher temperatures was accompanied by an increase in the size of green-synthesized nanoparticles. Subsequently, IONPs-G were dispersed in a mixture of solvent containing Tw80 to form a stable IONPs-GTw80 (XRD, FTIR, and XPS of IONPs-GTw80 powder were presented in Figures S1–S3 in Supplementary File). The UV–visible spectrophotometric analysis of the colloidal dispersion of IONPs-GTw80 versus IONPs-G in DDW (Figure S4 in the Supplementary File) revealed that the absorption peak of IONPs-GTw80 was observed at a lower wavelength (294.5 nm) than IONPs-G (325 nm). This could be linked to the smaller particle size for IONPs-GTw80. This is consistent with the literature, where a blue shift of UV–visible spectrophotometric analysis was linked to the smaller particle size of nanoparticles [47].

DLS analysis further confirmed the smaller particle size of IONPs-GTw80 (9.7 ± 2.1 nm) and its less tendency to aggregate (PDI, 0.111), indicating its narrow size distribution [46], and its homogeneity. Furthermore, IONPs-GTw80 was shown to be more stable with less tendency to aggregate in PBS (10 mM, pH 7.4) than IONPs-G as revealed from particle size and PDI values measured at all time points (Table S2, and Figure S5 in Supplementary File). The marked improvement of the key physicochemical properties (particle size and PDI) of IONPs-GTw80 could be linked to the dispersion of nanoparticles’ powder in ethanol, where the presence of ethanol as a wet medium was reported to reduce the possibility of particle adhesion and therefore, prevents aggregation [49,50,51,52,53,54,55]. This allows the particles to be subjected to a greater mechanical force upon stirring and facilitates the effective crushing of the powder and, therefore, reduction of particle size. In addition, ethanol might act as a dispersing agent to provide uniform distribution of particles and decrease the incidence of particle aggregation. Additionally, the lower tendency of IONPs-GTw80 to aggregate could be linked to steric stabilization effect of Tw80 molecules that are likely to be adsorbed onto the surface of IONPs-G. Despite the relatively low zeta potential values recorded for IONPs-GTw80 at all time intervals compared to IONPs-G (Table S2 in Supplementary File), the steric hindrance offered by Tw80 could overcome the attractive forces between particles, thereby maintaining dispersion stability and resulting in a lower PDI value [35,56,57,58,59].

Zeta potential measurements are often used to predict the stability of nanoparticle colloidal dispersions, where particles are less likely to aggregate if zeta potential values range from 30 to 60 mV [60,61,62,63,64,65,66]. Particles tend to aggregate significantly if the zeta potential is ≤5 mV [67,68]. However, it has been reported that this rule of thumb cannot strictly be applied to nanoparticle colloidal dispersion containing steric stabilizers. The adsorption of steric stabilizer onto nanoparticles might reduce the zeta potential due to the shift in the shear plane of the particle [69], but it is still able to overcome the attraction forces between particles, reducing the incidence of particle collisions, and therefore, the incidence of aggregate formation [46]. In other words, the zeta potential value could be less than 30 mV, but steric stabilizers are still able to prevent aggregation of nanoparticles in colloidal dispersion [70]. Furthermore, steric stabilization has been shown to have several advantages over electrostatic stabilization, for example, addition of electrolytes into sterically stabilized nano-systems doesn’t influence their stability [71,72], and nanoparticles that are sterically stabilized can be easily re-dispersed into the system [73,74]. The obtained data is consistent with a previous study where Tw80 was reported to improve gold nanoparticle stabilization and prevent their aggregation [34] through forming a thicker layer around the surface of gold nanoparticles [75]. Another study [76] also reported stabilization of silver nanoparticles by Tween 80 over 6 months without marked changes in the particle size over 6 months storage, although zeta potential of silver nanoparticles was −1 ± 2 mV.

Furthermore, ethanol–water mixtures were reported to exhibit higher viscosity compared to the pure solvents, which may enhance stability by reducing particle collision frequency [77,78]. Additionally, the presence of Tw80 contributed to the increased medium viscosity, thereby further decreasing the likelihood of particle collisions.

The TEM image showed a spherical shape for IONPs-GTw80 but with a lower particle size than what was detected by DLS. DLS is unreliable when measuring the size of individual particles in aggregates [7,35] as the hydrodynamic radius determined by DLS can be significantly larger than sizes measured using TEM [30,79]. However, in the current study, smaller differences in particle size were observed between DLS and TEM compared to the difference recorded for IONPs-G in our previous publication [30]. This might be attributed to the lower tendency of IONPs-GTw80 to aggregate in the presence of Tw80/ethanol–water mixture as previously discussed. HRTEM image (Figure S1C) showed the spherical shape of IONPs-GTw80 and implied its amorphous nature [80] that is consistent with the data obtained from XRD analysis (Figure S1A,B).

Concerning the antibacterial study, the plant extract did not show any antibacterial activity against the tested bacteria and this is consistent with our previous publication [30]. Quercus sp. Galls are reported [81] to be enriched with polyphenolic compounds that might show effective antibacterial activity; however, the lack of antibacterial activity observed in the current study might be linked to employing a resistant bacterial strain in the current study (Table S5 in the Supplementary File).

The ten-fold improvement of antibacterial activity observed with IONPs-GTw80 over IONPs-G against tested resistant bacteria might be attributed to its lower particle size (9.7 nm) and reduced propensity of particles to aggregate (PDI, 0.111). This is consistent with the literature [82,83], where nanoparticles with smaller sizes are characterized to have a greater surface area compared to aggregated particles. This might be associated with more effective interactions with bacterial cells and therefore greater antibacterial effect. This could explain the lower MIC and MBC values observed for IONPs-GTw80 compared to IONPs-G. However, MIC and MBC values of IONPs-GTw80 are still slightly higher than those recorded in our previous publication [30] for IONPs synthesized by the chemical co-precipitation method using cetyltrimethylammonium bromide (CTAB) as a capping agent to stabilize IONPs. This is consistent with the literature, where silver nanoparticles showed a lower antibacterial activity against *S. aureus* and *E. coli* when Tween 80 was used as a stabilizer than those stabilized with CTAB [84]. This might be linked to the nature of compounds adsorbed onto the surface of IONPs, as when the surface of nanoparticles is functionalized with aromatic functional groups, amino groups, it can increase the likelihood of nanoparticles interacting with bacterial cells compared to COOH and OH functional groups [85]. CTAB is a cationic surfactant that has an amino group in its chemical structure and this might be associated with a more effective interaction with bacteria, which can explain its lower MIC and MBC values compared to IONPs-GTw80.

The antibacterial results obtained are consistent with a previous study [86] where IONPs derived from plant extracts (clove and green coffee extracts) showed marked antibacterial activity against sensitive strains of *S. aureus* and *E. coli*. However, the MIC value of clove-derived IONPs against *S. aureus* was 62.5 µg/mL, which is lower than the MIC value observed for IONPs-GTw80 in our study against drug-resistant bacterial strains. In contrast, a higher MIC value was identified against *E. coli* for the green-coffee-derived IONPs compared to the current study. This difference might be linked to strain variability, which can influence the bacterial response to different antimicrobial agents [83]. It is worth noting that MIC and MBC values reported by Mohamed and colleagues [86] were identical for each bacterial strain tested. To investigate the relationship between the key physicochemical properties of IONPs and their antimicrobial activity, Mohamed and his colleagues measured the particle size by TEM, while the polydispersity of nanoparticles was assessed by ImageJ, a freeware Java-based Image Processing Program instead of employing DLS analysis. The obtained results showed two distinctive peaks for each type of IONPs: for IONPs produced with clove extract, the peaks were observed at 50 nm and 175 nm, whereas those synthesized using green coffee extract showed peaks at 55 nm and 225 nm. The larger particle size observed with green-coffee-derived IONPs than that of clove-derived ones could be linked to the higher MIC/MBC values recorded with green-coffee IONPs against *S. aureus* and *E. coli*. However, this approach is considered less accurate for representing sample polydispersity compared to the DLS technique conducted in the present study.

Another study conducted by Dowlath and colleagues [87] reported that IONPs synthesized using plant extract had a particle size of 20.9 nm (measured by DLS in DDW), which is more than double the size of IONPs-GTw80 produced in the current study (9.7 nm). Despite the larger size, these nanoparticles still have antibacterial activity against Gram-negative bacteria, namely, *E. coli*, *P. aeruginosa*, and *K. pneumoniae* at a concentration of 100, 100, and 200 μg/mL, respectively. Although Dowlath and colleagues [87] did not mention the antibiotic susceptibility profile of the bacterial strains used in their study; the tested bacteria were reported to be sensitive to chloramphenicol, which was applied as a positive control. In contrast, the bacterial strains tested in our study were resistant to chloramphenicol. This difference in antibiotic susceptibility might explain the lower effective concentration required to eradicate *E. coli* in their study compared to the current study (Table S5, Supplementary File).

Another study [88] revealed a higher MIC value than the values observed in the current study, where biologically synthesized IONPs exhibited MIC values of 1500 µg/mL against resistant Gram-positive bacteria, namely *B. subtilis* (MTCC 441) and *S. haemolyticus* (MTCC 3383). For resistant Gram-negative bacteria, such as *S. enterica* (MTCC 111) and *E. aerogenes* (MTCC 8767), the MIC values were 1200 µg/mL and 500 µg/mL, respectively. Notably, IONPs biologically synthesized by Ingle and colleagues [88] revealed a particle size (DLS) of 83.3 nm, which is larger by more than eight times the size of IONPs-GTw80 produced in the current study (9.7 nm). This difference in size may contribute to the enhanced interaction of IONPs-GTw80 with bacterial cells, thereby explaining the lower MIC and MBC values observed for IONPs-GTw80 in the present study.

On the other hand, study [89] demonstrated the antibacterial activity of Fe_2_O_3_ nanoparticles biologically synthesized using the cell-free bacterial supernatant of *E. coli* S1B. The Fe_2_O_3_ NPs had MIC values of 45, 40, and 20 µg/mL against *B. cereus* ATCC 6633, *S. aureus* ATCC 25923, and *P. aeruginosa* ATCC 27853, respectively. As revealed, all MIC values were lower than the values observed in the current study. This could be explained by use of standard laboratory control bacterial strains that are well-known for their susceptibility to different antibiotics [90,91,92]. In contrast, the present study focused on multidrug-resistant strains, which likely contributed to the higher MIC values observed. From the above findings, it could be concluded that the particle size and degree of particle aggregation are key physicochemical parameters governing the antibacterial activity of metal-based nanoparticles.

Concerning the time–kill curve study, the complete eradication of bacteria was recorded after 4 h, indicating that IONPs-GTw80 had a bactericidal activity. Typically, an antimicrobial agent is classified as a bactericidal agent if it kills 90% of the initial inoculum after 6 h [39]. Moreover, a compound is classified as a bactericidal agent when the ratio between MBC and MIC is less than or equal to four [93]. Herein, the MBC value of IONPs-GTw80 was found to be twice the MIC for both tested bacterial strains, which confirmed their bactericidal activity. The potent bactericidal activity of IONPs-GTw80 may be linked to their small particle size, which provides a larger surface area and consequently enhances the likelihood of interaction with bacterial cells, leading to cell death.

According to the authors’ knowledge, there are no studies in the literature that investigate the time–kill kinetics of green-synthesized IONPs, and correlate them with their key physicochemical properties. For instance, Kunjan and colleagues reported that IONPs synthesized using stem extract of *Coleus amboinicus* reduced the initial population of *S. mutans* and *C. albicans* by 50% after 4 h of incubation with 100 µg/mL of the nanoparticles [94]. However, it is important to mention that Kunjan and colleagues neglected to report the particle size, PDI, and zeta potential of IONPs; they failed to correlate these key physicochemical properties with the outcomes observed in their time–kill curve study. Another study performed by Govindharaj and colleagues [95] reported a time–kill study using hydrocotyle umbellata mediated IONPs against urinary tract pathogens; *S. aureus* and *E. coli*. The IONPs produced showed minimal bactericidal and bacteriostatic effects at a concentration of 100 µg/mL, reducing bacterial growth from 5.5 log cfu/mL to 4 log cfu/mL for both types of bacteria. Again, Govindharaj and colleagues did not report the particle size, PDI, or zeta potential values of the IONPs, thus failed to correlate these critical physicochemical properties with the results of their time–kill curve study.

TEM images presented in Figure 3 and Figure 4 demonstrated the complete bacterial damage following treatment with IONPs-GTw80. Previously, we reported [30] that the antibacterial mechanism of IONPs-G was linked to the generation of reactive oxygen species (ROS), with the level of ROS being concentration-dependent. ROS release may contribute to DNA damage, disruption of the bacterial cell wall integrity, and damage to bacterial cell membranes [86,96,97,98]. However, TEM in the current study (Figure 3 and Figure 4) suggested that the physical damage may also be another mechanism contributing to the antibacterial activity of IONPs-GTw80 and this could be related to the small particle size of IONPs-GTw80 (9.7 nm). TEM images revealed a significant accumulation of IONPs-GTw80 on the surface of both types of bacteria, which may lead to the disruption of bacterial cell membranes and the release of cytoplasmic contents. The small particle size of IONPs-GTw80 may make their entry into the cytoplasm easier, which, combined with ROS release that damages cell organelles and leads to vacuole formation. This is consistent with other studies [99,100]. Li and colleagues [99] reported that IONPs adsorbed onto the surface of *E. coli*, physically harming the outer membrane, which in turn causes leakage of the cytoplasmic content. Furthermore, TEM revealed multiple stages of IONPs internalization in *E. coli*, starting from surface adsorption to cytoplasmic accumulation, ultimately leading to formation of intracellular vacuoles. As a result, they concluded that the bactericidal effect of IONPs is a result of both physical damage and ROS release.

Another study by Simon-Deckers and colleagues [101] reported that both physical damage and ROS production contributed to the antibacterial activity of Al_2_O_3_ NPs and TiO_2_ NPs against *E. coli* [101]. They reported that these NPs adhered to the cell wall of bacteria (*C. metallidurans* CH3 and *E. coli* MG1655), followed by internalization into the periplasm, leading to bacterial toxicity. Furthermore, exposure to these nanoparticles resulted in an increase in cellular ROS, which contributed to the damage of intracellular organelles and ultimately caused bacterial death. Similar observations were also reported by Kumar and colleagues [102], where TEM images of *E. coli* treated with ZnO NPs and TiO_2_ NPs showed nanoparticles adhering to the bacterial surface and uniformly distributing inside the cells, accompanied by ROS production. Kumar and colleagues concluded that both ROS generation and physical damage were responsible for bacterial cell death.

Due to the small size of nanomaterials, they have a high potential to penetrate the skin and eyes upon topical application, resulting in their accumulation in the biological system, which may cause adverse effects. Therefore, it was critical to conduct an in vivo cytotoxicity study of IONPs-GTw80 at its MBC. Gross examination and histopathological investigations showed that IONPs-GTw80 was compatible with the animals’ eyes and skin. This is likely linked to IONPs’ concentration-dependent release of ROS, as demonstrated in our previous study [30]. In other words, a lower MBC value of IONPs-GTw80 than IONPs-G could be linked to lower level of ROS release, resulting in a lower cytotoxic effect. In addition, the coating of nanoparticles with surfactants or co-surfactants was reported to alter the surface characteristics of the particles, which in turn affects their physicochemical properties. This modification may contribute to the sustained release of metal ions, thereby reducing the toxicity profile of the coated nanoparticles [103,104]. Other studies reported that IONPs coated with dextran and the polymer polyethylene glycol (PEG) demonstrated reduced cytotoxicity compared to their uncoated counterparts. In addition to lower toxicity, the coated nanoparticles were found to better preserve cell morphology compared to the uncoated particles [105].

Biochemical analysis was carried out to measure liver enzyme levels and assess the potential accumulation of IONPs-GTw80 in the liver after topical application. The liver is the main organ for nanomaterial accumulation, and this might be associated with an increase in the levels of liver enzymes [106]. The results obtained in this study indicated that the topical application of IONPs-GTw80 was non-toxic to the liver, as there were no statistically significant (*p* > 0.05) differences in liver enzymes (ALT and ALK) in the treated groups when compared to the untreated group. This finding aligns with our previous publication [30], where the topical application of IONPs synthesized by green and chemical methods was not associated with any harmful effect on the liver. Another study reported that the topical application of copper nanoparticles for wound healing did not result in any liver-related adverse effects, as there were no changes in liver enzyme levels in the treated animals compared to the untreated ones, suggesting the safety of the nanomaterials upon topical application [107]. Hematological analysis was aligned with our previous publication [30], where topical application of IONPs-GTw80 was not associated with any signs of adverse effects and this assured the biocompatibility and safety of IONPs-GTw80. Therefore, IONPs-GTw80 may be recommended as a potential topical antimicrobial agent and/or disinfectant for use in healthcare settings, as well as an antimicrobial agent for treatment of infected wounds. Nonetheless, for the clinical translation of IONPs-GTw80, additional studies are still required to fully evaluate its antimicrobial efficacy, wound healing capacity for both infected and non-infected wounds, and safety profile in humans.

## 5. Conclusions

The objective of the present study is to modulate the key physicochemical properties of green-synthesized iron oxide nanoparticles (IONPs-G) to enhance their antibacterial activity and biosafety. Instead of extensively investigating synthesis parameters, a post-synthesis approach was employed by dispersing IONPs-G in a mixture of solvent containing Tween 80 (Tw80), which effectively stabilized the nanoparticles via the steric stabilizing effect. The formed nanoparticles (IONPs-GTw80) had an average particle size of 9.7 ± 2.1 nm, with a polydispersity index of 0.111 ± 0.02, and a zeta potential of −11.4 ± 2.4 mV. This strategy significantly improved their antibacterial activity, where the MIC value against *S. aureus* and *E. coli* was reduced by more than ten-fold compared to IONPs-G. Additionally, MBC was twice MIC, suggesting the bactericidal activity of IONPs-GTw80.

Moreover, in vivo studies demonstrated the biocompatibility of IONPs-GTw80 as they were well tolerated on rabbit skin and eyes, and this was further supported by histopathological and biochemical analyses.

This study presents a simple, effective, scalable, and eco-friendly method for modulating the physicochemical properties of green-synthesized IONPs post-production. This further emphasized the idea that both the antimicrobial activity and biosafety of nanomaterials are primarily dependent on their key physicochemical characteristics rather than the synthesis methodology. Given their enhanced antimicrobial properties and biocompatibility, IONPs-GTw80 showed a strong potential as disinfectants or topical antimicrobial agents for healthcare applications, including management of infected wounds, though in vivo tests to address their wound healing capacity as well as long-term safety evaluations are still required for their clinical translation.

## Figures and Tables

**Table 1:** Key physicochemical properties of IONPs-GTw80, including particle size, polydispersity index (PDI), and zeta potential measurements. Data presented as an average ± standard deviation (SD). Results are an average of three replicates.

Sample Name	Particle Size (D nm ± SD)	PDI ± SD	Zeta Potential (mV ± SD)
IONPs-GTw80	9.7 ± 2.1	0.111 ± 0.02	−11.4 ± 2.4

**Table 2:** MIC and MBC * (µg/mL) values of IONPs-GTw80 recorded for *S. aureus* and *E. coli.*

Sample	*S. aureus*		*E. coli*	
	MIC	MBC	MIC	MBC
** Plant Extract	NS	NS	NS	NS
** Blank sample of IONPs-GTw80	NS	NS	NS	NS
IONPs-GTw80	100	200	100	200

* MIC: Minimum inhibitory concentration, MBC: Minimum bactericidal concentration. ** NS = resistant bacteria tested in the current study were non-sensitive (NS) to the plant extract, and the blank sample of IONPs-GTw80.

**Table 3:** * Effect of IONPs-GTw80, and its blank solution, on serum biochemical markers compared to untreated animals. The results were expressed as mean ± standard error (SE) with three independent measurements conducted per sample.

Animal Group	ALK (U/L) ± SE	ALT (U/L) ± SE
Untreated animal (Negative control)	31.02 ± 2.35	28.45 ± 1.75
IONPs-GTw80 (200 µg/mL)	31.67 ± 2.85	27.98 ± 2.43
Blank solution	30.91 ± 2.55	28.32 ± 2.71

* Two-way ANOVA followed by the Tukey post hoc test for comparisons between multiple groups at a confidence level of 95% was used for data analysis. A non-significant (*p* > 0.05) difference between untreated (control) and treated animals was identified. (U/L) = Unit/Liter. Alanine aminotransferase (ALT) and alkaline transferases (ALKs).

**Table 4:** * Effect of IONPs-GTw80, and its blank solution, on the animal erythrogram compared to untreated animals. The results were expressed as mean ± standard error (SE) with three independent measurements conducted per sample.

Animal Groups	PCV (%) ± SE	Hb (g/dL) ± SE	RBCs (×10^6^/nL) ± SE	MCV (fL) ± SE	MCHC (%) ± SE
Untreated animal (Negative control)	39.48 ± 2.96	11.00 ± 2.84	8.92 ± 0.21	48.60 ± 0.23	38.26 ± 2.46
IONPs-GTw80 (200 µg/mL)	38.57 ± 0.85	11.85 ± 0.92	7.85 ± 0.58	48.90 ± 3.85	39.85 ± 5.85
Blank solution	37.41 ± 1.26	12.34 ± 2.21	8.61 ± 1.15	47.92 ± 1.25	38.27 ± 1.23

* Two-way ANOVA followed by the Tukey post hoc test for comparisons between multiple groups at a confidence level of 95% was used for data analysis. A non-significant (*p* > 0.05) difference between untreated (control) and treated animals at *p* < 0.05 was identified. PCV = packed cell volume, Hb = hemoglobin (g/dL = gram/deciliter), RBCs = red blood cells’ count, MCV = main corpuscular volume (fL, femto-liter), MCHC = main corpuscular hemoglobin concentration.

## Data Availability

Data presented in this study is contained within the article and supplementary material. Further inquiries can be directed to the corresponding author.

## Supplementary Materials

The following supporting information can be downloaded at: https://www.mdpi.com/article/10.3390/pharmaceutics17111371/s1, Table S1: LC-MS/MS analysis of some phenolic metabolites from aqueous extract of Quercus infectoria galls (QIGs); Figure S1: XRD spectra of IONPs-G, IONPs synthesized by plant extract in absence of Tween 80 (A), and IONPs-GTw80, IONPs synthesized by plant extract in presence of Tween 80 (B), high-resolution transmission electron microscopy (HRTEM) of IONPs-GTw80 (C); Figure S2: FTIR spectra of gall powder, IONPs-G, IONPs synthesized by plant extract in the absence of Tween 80 and IONPs-GTw80, IONPs synthesized by plant extract in the presence of Tween 80; Figure S3: XPS analysis for IONPs-G and IONPs-GTw80; Figure S4: UV–visible spectra of plant extract (blue line), IONPs-G, IONPs-GTw80 synthesized by plant extract in the presence of Tween 80 (red line) and IONPs-G (green line), IONPs synthesized by plant extract in the absence of Tween 80; Table S2: Stability of IONPs-G versus IONPs-GTw80 dispersed in PBS; Figure S5: Particle size of IONPs-G (A) versus IONPs-GTw80 (B) dispersed in PBS; Table S3: Skin irritation assessment after treatment with IONPs-GTw80 colloidal dispersion and its blank solution compared to untreated animals; Table S4: Assessment of eye irritations following eyes treatment with IONPs-GTw80 and its blank solution versus untreated eyes; Table S5: Antibiotic susceptibility profile of tested bacteria (*S. aureus* and *E. coli*) according to CLSI. References [30,38,47,80,81,86,108,109,110,111,112,113,114,115,116,117,118,119,120,121,122,123,124,125] are cited in Supplementary Materials.
