# Supplementary material for: Post-Synthesis Modulation of the Physicochemical Properties of Green-Synthesized Iron Oxide Nanoparticles with Tween 80 to Enhance Their Antibacterial Activity and Biocompatibility
Source: Pharmaceutics. 2025 Oct 23;17(11):1371. doi: 10.3390/pharmaceutics17111371 (PMC12655375; doi:10.3390/pharmaceutics17111371)
Supplement: Supplementary file 1 [file pharmaceutics-17-01371-s001.zip › pharmaceutics-3825954-supplementary.pdf]

Supplementary file

Table S1: LC-MS/MS analysis of some phenolic metabolites from aqueous extract of Quercus infectoria galls (QIGs)				
**Number**	***Rt**	**Supposed compound**	**$[M-H]^-$**	**Reference**
**1**	0.932	Quinic acid	191	[1]
**2**	1.024	Gallic acid	169	[1]
**3**	1.138	Monogalloyl glucose	331	[1]
**4**	1.275	Dihydroxy benzoic acid	153	[1]
**5**	1.339	2-*O*-galloyl hydroxymalonic acid	271	[2]
**6**	1.44	Galloyl glyceride	243	[1]
**7**	1.725	Digalloyl glucose I	483	[1]
**8**	1.998	Digalloyl glucose II	483	[1]
**9**	2.298	Methyl gallate	183	[1]
**10**	2.817	Theogallin	343	[2]
**11**	3.599	Trigalloyl glucose I	635	[1]
**12**	3.856	Trigalloyl glucose II	635	[1]
**13**	4.7	Tellimagrandin I	785	[2]
**14**	4.873	Tetra galloyl glucose	787	[1]
**15**	5.133	Syringic acid	197	[1]
**16**	5.319	Epicatechin	289	[3]
**17**	7.414	Penta galloyl glucose	939	[1]
**18**	7.749	Ellagic acid	301	[1]
**19**	7.957	Eugeniin	937	[2]
**20**	10.606	Luteolin	285	[4]
***Rt: retention time**				

For more details about the composition of plant extract, readers are directed to our previous publication [5]

Supplementary file

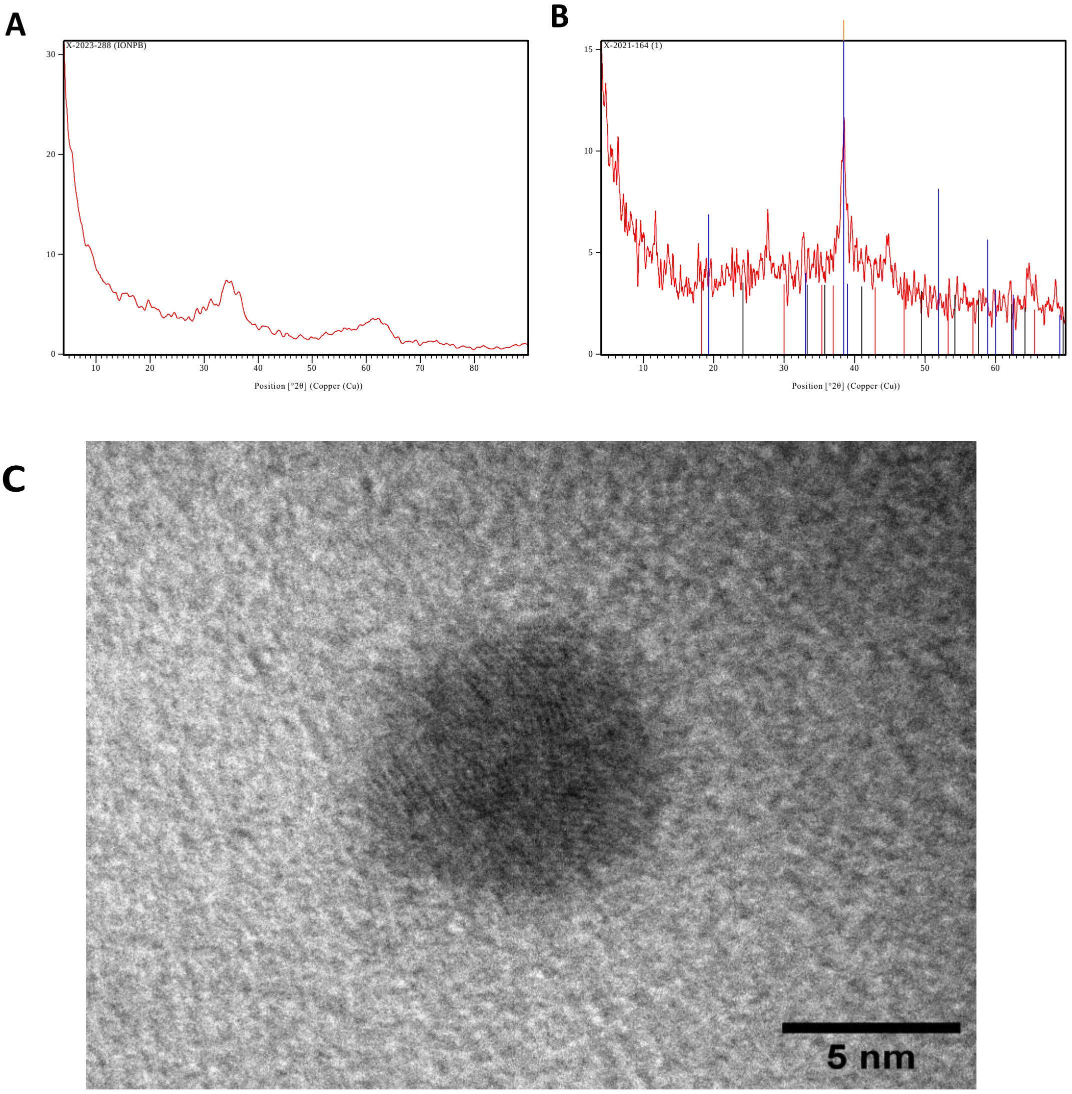

Figure S1: XRD spectra of IONPs-G, IONPs synthesized by plant extract in absence of Tween 80 (A), and IONPs-GTw80, IONPs synthesized by plant extract in presence of Tween 80 (B), high-resolution transmission electron microscopy (HRTEM) of IONPs-GTw80 (C).

IONPs-G and IONPs-GTw80 showed weak diffraction peaks, indicating their amorphous nature [6–8]. For IONPs-G (Figure S1A), broad peak was found starting at 2ϴ: 28 and ending at 2ϴ: 42, and another broad peak starting at 2ϴ: 52 and ending at 2ϴ: 67. This is consistent with the literature [6,9–13] that previously reported XRD spectra of Fe_2O_3 nanoparticles synthesized by plant extract. For IONPs-GTw80 (Figure S1B), characteristic peak found at 2ϴ: 38.4 is consistent with the literature [14] or IONPs (Fe_2O_3) coated with Tween 80. HRTEM image of IONPs-GTw80 (Figure S1C) further confirmed its amorphous nature [15].

Supplementary file

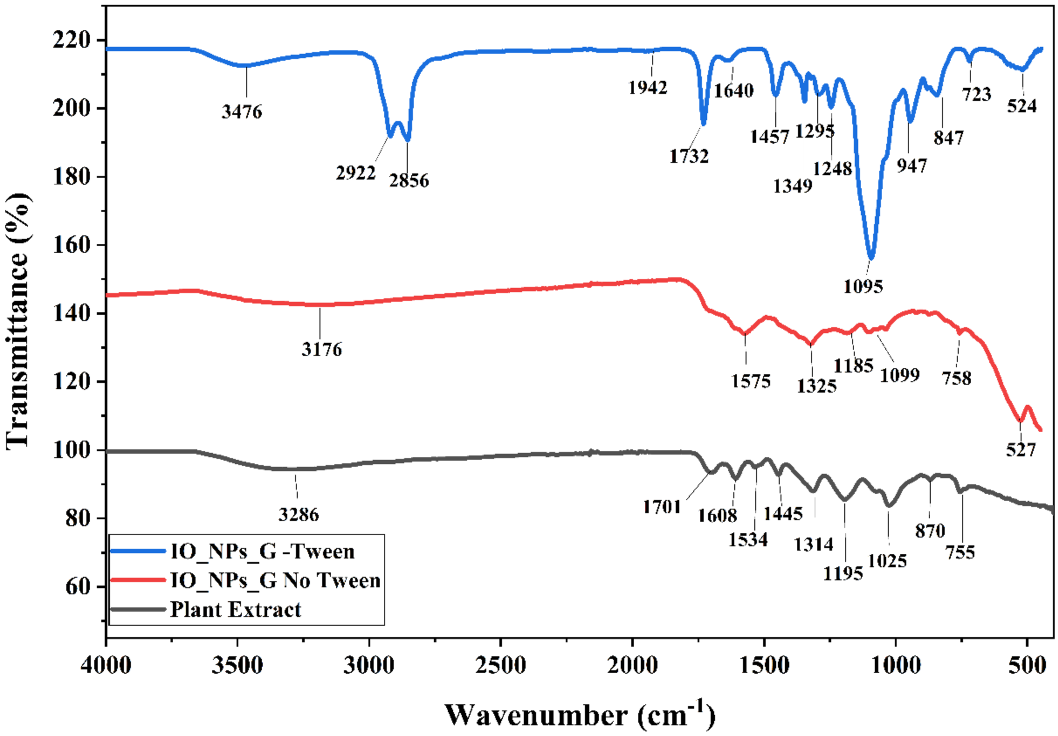

Figure S2: FTIR spectra of gall powder, IONPs-G, IONPs synthesized by plant extract in the absence of Tween 80, and IONPs-GTw80, IONPs synthesized by plant extract in the presence of Tween 80.

In the IONPs-G spectrum (IONPs-G synthesized in the absence of Tween 80), a prominent absorption band at 527 cm^{-1} was identified, indicating the presence of an Fe–O bond. The minor peaks observed at 758 cm^{-1} and 1099 cm^{-1} could be attributed to the stretching vibration modes of the Fe-O group. This is consistent with our previous publication [5], confirming the successful synthesis of IONPs-G. A slight shift of those peaks (524 and 723 cm^{-1}) was observed for IONPs-GTw80 (IONPs-G prepared in the presence of Tween 80), which could be correlated to the adsorption molecules of Tween 80 on the surface of IONPs-G. The nanoparticles synthesized in the presence of Tween-80 have the following characteristic peaks of Tween 80: CH_2 (2856 cm^{-1}), CH_3 (2922 cm^{-1}), C–O–C (1098 cm^{-1}), and C=O stretch at 1732 $cm^{-1,}$ and this confirms the absorbance of Tween 80 molecules onto the surface of IONPs [14]. The broad band observed in the spectrum of IONPs-GTw80 at 3476 cm^{-1} could be linked to OH groups of Tween 80 and the O–H stretching vibration of phenolic groups of plant extract [5].

For more details and explanation about other peaks that appeared in the FTIR spectrum, readers are kindly directed to check our previous publication [5].

<u>**Supplementary file**</u>

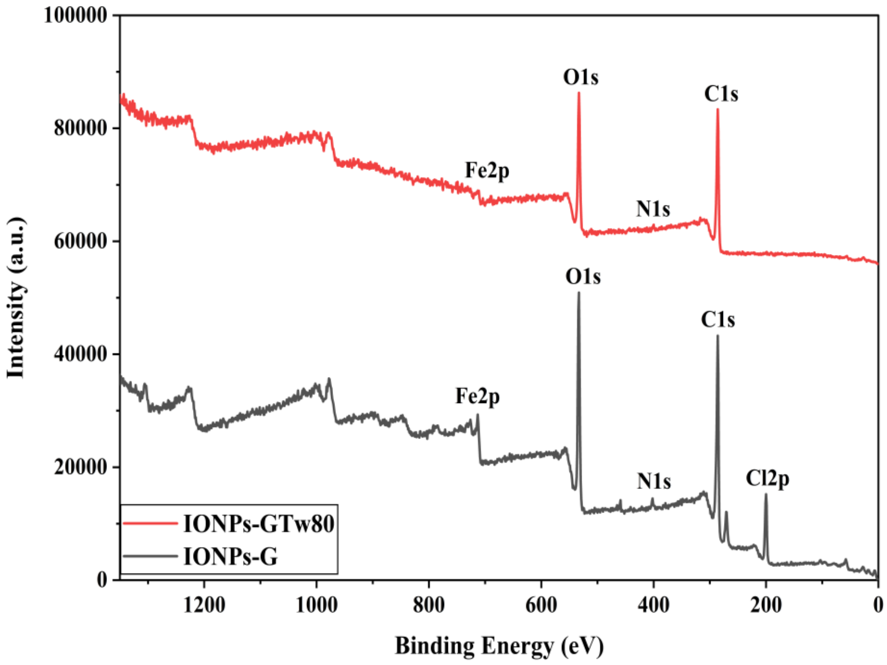

Figure S3: XPS analysis for IONPs-G and IONPs-GTw80

Figure S3 showed the XPS spectrum for IONPs-G that was identical to our previous publication [5] and to the spectrum of IONPs-GtW80. XPS analysis was conducted to explore the oxidation state of iron (Fe) and oxygen (O) present in IONPs, and their elemental analysis. The XPS spectrum revealed the presence of several elements, including Fe, O, N, and C. The photoelectron peaks at 533.29 eV indicated the presence of oxygen (O) on the surface of IONPs-G/IONPs-GTw80, associated with O–H, O–C, and O=C compounds originating from the plant extract used in the synthesis of IONPs as well as Tween 80 molecules adsorbed onto the surface [16]. Additionally, these peaks correspond to the oxygen found in Fe_2O_3 [17]. Carbon (C) signals at 200.13 and 286.1 eV affirmed the existence of alkyl or aliphatic compounds within the biomolecules of the plant extract and Tween 80 molecules. Furthermore, the peaks at 712.97 eV served as a distinctive signature of Fe 2p, specifically indicating the presence of Fe^{3+} ion [18]. These $Fe3^{+}$ ions are associated with α-Fe2O3 [19]. The core levels of Fe 2p and O 1s exhibited oxidation states of +3 and + 2, respectively, confirming that the nanoparticles formed are pure α-Fe2O3 and do not contain impurities such as zero-valent iron or other forms of iron oxide [18]. The XPS survey spectra showed a peak at binding energies 712.39 eV that is linked to signal of Fe2P, corresponding to Fe3+ in α-Fe_2O_3 NPs; this peak is consistent with the oxygen signal at binding energy 531.39 eV recorded for IONPs-G/IONPs-GTw80 [20,21]. In addition, the presence of an insignificant amount of nitrogen, carbon, and chlorine within the α-Fe2O3 sample (IONPs-G) may be linked to the preparation process [20,21].

Supplementary file

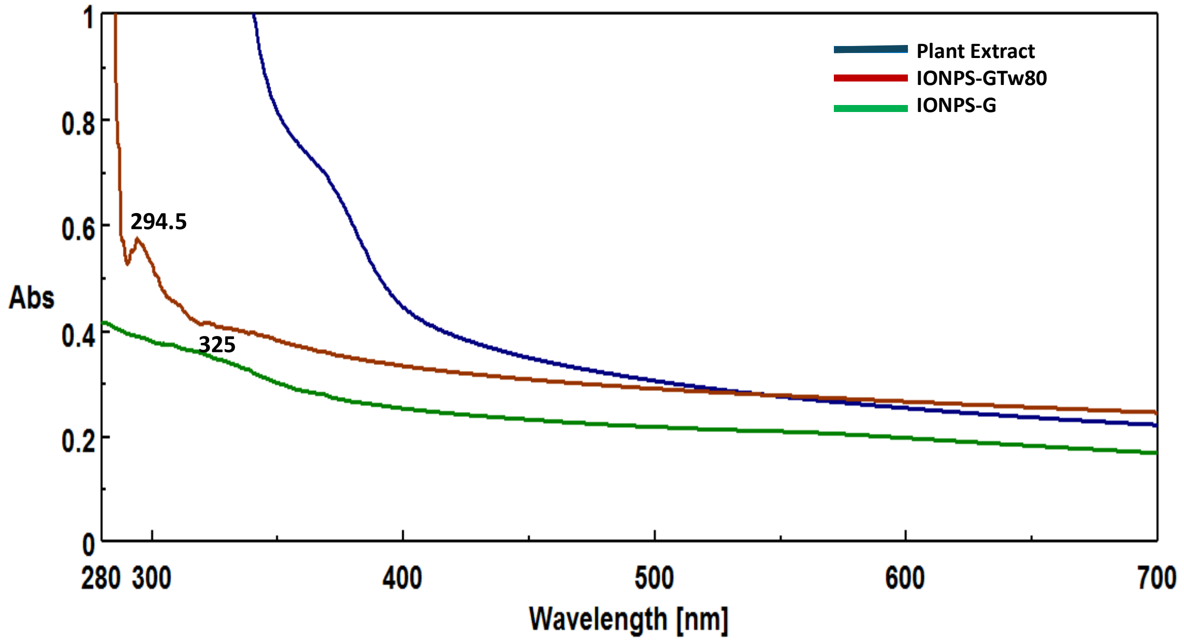

Figure S4: UV–visible Spectra of plant extract (blue line), IONPs-G, IONPs-GTw80 synthesized by plant extract in the presence of Tween 80 (red line), and IONPs-G (green line), IONPs synthesized by plant extract in the absence of Tween 80.

UV–visible spectrophotometric analysis of IONPs-G and IONPs-GTw80 presented in Figure S4 showed an absorption peak at 325 and 294.5 nm, respectively. This is consistent with the absorption spectrum of Fe_2O_3 nanoparticles reported in the literature [22]. The blue shift of the absorption peak from 325 nm (IONPs-G) to 294.5 nm (IONPs-GTw80) could be linked to the smaller particle size of IONPs-GTw80 compared to IONPs-G [23].

Supplementary file

Table S2: Stability of IONPs-G versus IONPs-GTw80 dispersed in PBS

Time (h)	Particle Size (D nm ± SD)	PDI ± SD	Zeta Potential (mV ± SD)
	**IONPS_G**		
**0**	620.900±36.549	0.480±0.081	-34.000±1.054
**1**	821.167±84.350	0.524±0.087	-32.933±1.002
**3**	887.967±87.875	0.575±0.074	-31.233±1.890
**6**	835.367±227.687	0.509±0.072	-32.100±0.400
**24**	797.600±170.622	0.485±0.122	-32.167±1.305
	**IONPS_GTw80**		
**0**	24.453±0.775	0.224±0.010	-18.200±1.311
**1**	12.037±0.031	0.215±0.002	-25.633±0.503
**3**	14.870±1.071	0.367±0.004	-26.533±1.626
**6**	14.637±0.463	0.328±0.009	-26.800±1.127
**24**	19.077±0.281	0.298±0.053	-25.867±0.306

PDI, polydispersity index

Supplementary file

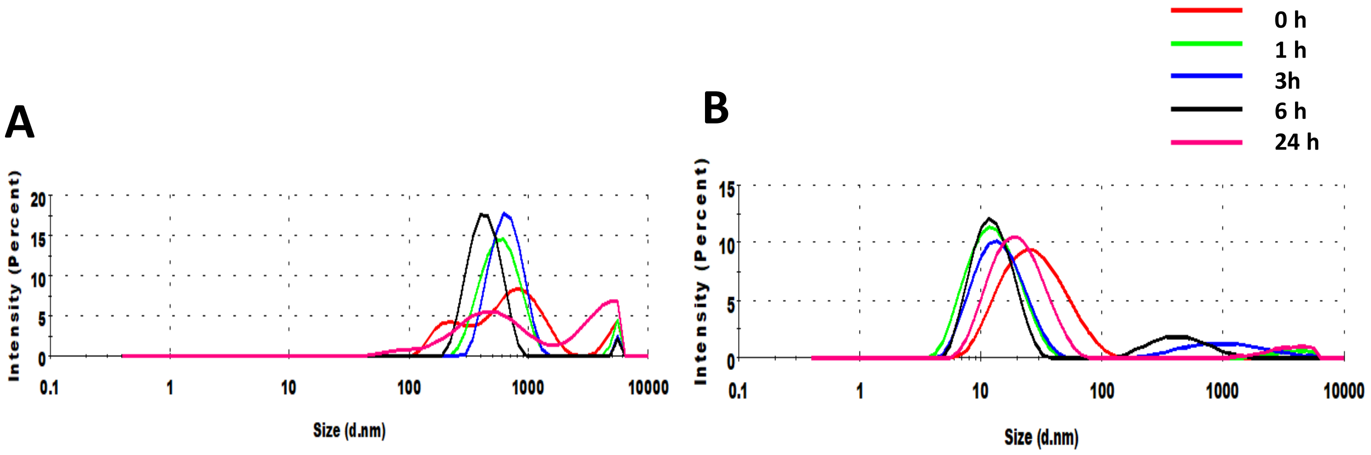

Figure S5: Particle size of IONPs-G (A) versus IONPs-GTw80 (B) dispersed in PBS

Supplementary file

Table S3: *Skin irritation assessment after treatment with IONPs-GTw80 colloidal dispersion and its blank solution compared to untreated animals.

		Control sits								Treated sits							
		**Erythema and Eschar**				**Edema**				**Erythema and Eschar**				**Edema**			
		**Intact**		**Abraded**		**Intact**		**Abraded**		**Intact**		**Abraded**		**Intact**		**Abraded**	
**Hours/Animals**		**24**	**72**	**24**	**72**	**24**	**72**	**24**	**72**	**24**	**72**	**24**	**72**	**24**	**72**	**24**	**72**
**Untreated animals**	1	0	0	0	0	0	0	0	0	0	0	0	0	0	0	0	0
	2	0	0	0	0	0	0	0	0	0	0	0	0	0	0	0	0
	3	0	0	0	0	0	0	0	0	0	0	0	0	0	0	0	0
	4	0	0	0	0	0	0	0	0	0	0	0	0	0	0	0	0
**IONP-GTw80 (200µg/ml)**	5	0	0	0	0	0	0	0	0	0	0	0	0	0	0	0	0
	6	0	0	0	0	0	0	0	0	0	0	0	0	0	0	0	0
	7	0	0	0	0	0	0	0	0	0	0	0	0	0	0	0	0
	8	0	0	0	0	0	0	0	0	0	0	0	0	0	0	0	0
**Blank**	9	0	0	0	0	0	0	0	0	0	0	0	0	0	0	0	0
	10	0	0	0	0	0	0	0	0	0	0	0	0	0	0	0	0
	11	0	0	0	0	0	0	0	0	0	0	0	0	0	0	0	0
	12	0	0	0	0	0	0	0	0	0	0	0	0	0	0	0	0

***All skin irritation scores were 0 after 24 and 72 h; both intact and abraded skin showed no symptoms of erythema, eschar, and edema, indicating the absence of irritation.

Supplementary file

Table S4: *Assessment of eye irritations following eye treatment with IONPs-GTw80 and its blank solution versus untreated eyes

Tested Solution	Tissues examined in the eye	Number of rabbits									
		1		2		3		4		5	
		RT. Untreated	LT. treated	RT. Untreated	LT. treated	RT. Untreated	LT. treated	RT. Untreated	LT. treated	RT. Untreated	LT. treated
**IONPs- GTw80 (200µg/ml)**	Cornea	0	0	0	0	0	0	0	0	0	0
	Iris	0	0	0	0	0	0	0	0	0	0
	Conjunctiva	0	0	0	0	0	0	0	0	0	0
**Blank solution**	Cornea	0	0	0	0	0	0	0	0	0	0
	Iris	0	0	0	0	0	0	0	0	0	0
	Conjunctiva	0	0	0	0	0	0	0	0	0	0

*All eye irritation scores were 0. The observations were concerned with corneal opacity, reactivity of iris, conjunctival edema, and ocular discharge. No symptoms of irritation were noted.

Supplementary file

Table S5: Antibiotic susceptibility profile of tested bacteria (*S. aureus* and *E. coli*) according to CLSI [24].

Bacterial strains	Chemical group of antibiotics (µg)																			
	Aminoglycoside		ß-lactams			Cephems								Glycopeptides	Quinolones		Sulfonamide	lincosamide	Macrolide	Phenicol
	GMN_{10}	KAN	AMP_{10}	P_{10}	OX_{30}	FOX_{30}	CRO_{30}	CFM_{5}	CEC_{30}	CFP_{75}	CFR_{30}	CXM_{30}	$CFS_{75/30}$	VA_{30}	NOR_{10}	OFX_{5}	SXT_{25}	DA_{15}	E_{15}	C_{30}
S. aureus	S	S	R	R	R	R	R	R	R	R	R	R	R	S	R	S	S	R	R	R
E. coli	R	S	R	R	nt	R	R	R	R	R	R	R	R	R	R	R	R	nt	nt	R
R; resistant, S; sensitive, nt; not tested.																				

List of antibiotic abbreviations			
GMN: Gentamycin	FOX: Cefoxitin	CFR: Cephalosporine	OFX: Oflatoxin
KAN: Kanamycin	CRO: Ceftriaxone	CXM: Cefuroxime	SXT: Trimethoprim sulfamethoxazole
AMP: Ampicillin	CFM: Cefixime	CFS: Cefoperazone/sublactam	DA: Clindamycin
P: Penicillin	CEC: Cefaclor	VA: Vancomycin	E: Erythromycin
OX: Oxacillin	CFP: Cefoperazone	NOR: Norfloxacin	

Supplementary file

References:

1. Attia, H.G.; Albarqi, H.A.; Said, I.G.; Alqahtani, O.; Raey, M.A.E.I. Synergistic Effect between Amoxicillin and Zinc Oxide Nanoparticles Reduced by Oak Gall Extract against Helicobacter Pylori. *Molecules* **2022**, *27*, 4559, doi:10.3390/molecules27144559.

2. Abdullah, A.R.; Hapidin, H.; Abdullah, H. Phytochemical Analysis of Quercus Infectoria Galls Extracts Using FTIR, LC-MS and MS/MS Analysis. *Res. J. Biotechnol.* **2017**, *12*, 55–61.

3. Zhang, M.; Vervoort, L.; Moalin, M.; Mommers, A.; Douny, C.; den Hartog, G.J.M.; Haenen, G.R.M.M. The Chemical Reactivity of (-)-Epicatechin Quinone Mainly Resides in Its B-Ring. *Free Radic. Biol. Med.* **2018**, *124*, 31–39, doi:10.1016/j.freeradbiomed.2018.05.087.

4. Liu, W.; Kong, Y.; Zu, Y.; Fu, Y.; Luo, M.; Zhang, L.; Li, J. Determination and Quantification of Active Phenolic Compounds in Pigeon Pea Leaves and Its Medicinal Product Using Liquid Chromatography-Tandem Mass Spectrometry. *J. Chromatogr. A* **2010**, *1217*, 4723–4731, doi:10.1016/j.chroma.2010.05.020.

5. Ali, A.M.; Hill, H.J.; Elkhouly, G.E.; Raya, N.R.; Tawfik, N.F.; Bakkar, M.R.; El-basaty, A.B.; Stamataki, Z.; Abo-zeid, Y. Green and Chemical Synthesis of Iron Oxide Nanoparticles : Comparative Study for Antimicrobial Activity and Toxicity Concerns. *J. Drug Deliv. Sci. Technol.* **2025**, *103*, 106434, doi:10.1016/j.jddst.2024.106434.

6. Mohamed, A.; Atta, R.R.; Kotp, A.A.; Abo El-Ela, F.I.; Abd El-Raheem, H.; Farghali, A.; Alkhalifah, D.H.M.; Hozzein, W.N.; Mahmoud, R. Green Synthesis and Characterization of Iron Oxide Nanoparticles for the Removal of Heavy Metals (Cd^{2+} and Ni^{2+}) from Aqueous Solutions with Antimicrobial Investigation. *Sci. Rep.* **2023**, *13*, doi:10.1038/s41598-023-31704-7.

7. Xiao, Z.; Yuan, M.; Yang, B.; Liu, Z.; Huang, J.; Sun, D. Plant-Mediated Synthesis of Highly Active Iron Nanoparticles for Cr (VI) Removal: Investigation of the Leading Biomolecules. *Chemosphere* **2016**, *150*, doi:10.1016/j.chemosphere.2016.02.056.

8. Kouhbanani, M.A.J.; Beheshtkhoo, N.; Taghizadeh, S.; Amani, A.M.; Alimardani, V. One-Step Green Synthesis and Characterization of Iron Oxide Nanoparticles Using Aqueous Leaf Extract of Teucrium Polium and Their Catalytic Application in Dye Degradation. *Adv. Nat. Sci. Nanosci. Nanotechnol.* **2019**, *10*, doi:10.1088/2043-6254/aafe74.

9. Lakshmnarayanan, S.; Shereen, M.F.; Niraimathi, K.L.; Brindha, P. One - Pot Green Synthesis of Iron Oxide Nanoparticles from Bauhinia Tomentosa : Characterization and Application towards Synthesis of 1, 3 diolein. *Sci. Rep.* **2021**, 1–13, doi:10.1038/s41598-021-87960-y.

10. Aida, M.S.; Alonizan, N.; Zarrad, B.; Hjiri, M. Green Synthesis of Iron Oxide Nanoparticles Using Hibiscus Plant Extract. *J. Taibah Univ. Sci.* **2023**, *17*, doi:10.1080/16583655.2023.2221827.

11. Abasali karaj abad, Z.; Nemati, A.; Malek Khachatourian, A.; Golmohammad, M. Synthesis and Characterization of RGO/Fe2O3 Nanocomposite as an Efficient Supercapacitor Electrode Material. *J. Mater. Sci. Mater. Electron.* **2020**, *31*, doi:10.1007/s10854-020-04062-7.

12. Bibi, I.; Nazar, N.; Ata, S.; Sultan, M.; Ali, A.; Abbas, A.; Jilani, K.; Kamal, S.; Sarim, F.M.; Khan, M.I.; et al. Green Synthesis of Iron Oxide Nanoparticles Using Pomegranate Seeds Extract and Photocatalytic Activity Evaluation for the Degradation of Textile Dye. *J. Mater. Res. Technol.* **2019**, *8*, doi:10.1016/j.jmrt.2019.10.006.

Supplementary file

13. Yekeen, M.O.; Ibrahim, M.; Wachira, J.; Pramanik, S. Green Synthesis and Characterization of Iron Oxide Nanoparticles Using Egeria Densa Plant Extract. *Appl. Biosci.* **2025**, *4*, 27, doi:10.3390/applbiosci4020027.

14. Khan, Y.; Durrani, S.K.; Siddique, M.; Mehmood, M. Hydrothermal Synthesis of Alpha Fe2O3 Nanoparticles Capped by Tween-80. *Mater. Lett.* **2011**, *65*, doi:10.1016/j.matlet.2011.04.068.

15. Chen, B.; Ten Brink, G.H.; Palasantzas, G.; Kooi, B.J. Size-Dependent and Tunable Crystallization of GeSbTe Phase-Change Nanoparticles. *Sci. Rep.* **2016**, *6*, doi:10.1038/srep39546.

16. Chen, M.; Wang, L.Y.; Han, J.T.; Zhang, J.Y.; Li, Z.Y.; Qian, D.J. Preparation and Study of Polyacryamide-Stabilized Silver Nanoparticles through a One-Pot Process. *J. Phys. Chem. B* **2006**, *110*, doi:10.1021/jp061134n.

17. Iacovita, C.; Stiufiuc, R.; Radu, T.; Florea, A.; Stiufiuc, G.; Dutu, A.; Mican, S.; Tetean, R.; Lucaciu, C.M. Polyethylene Glycol-Mediated Synthesis of Cubic Iron Oxide Nanoparticles with High Heating Power. *Nanoscale Res. Lett.* **2015**, doi:10.1186/s11671-015-1091-0.

18. Ramananda, H.S.M.; Senthil, B.P.; Kumar, N.M.; Selvaraj, R. Structural Characterization of Cuboidal α-Fe_2O_3 Nanoparticles Synthesized by a Facile Approach. *Appl. Nanosci.* **2023**, *13*, 5605–5613, doi:10.1007/s13204-023-02780-y.

19. Shen, S.; Zhou, J.; Dong, C.; Hu, Y.; Tseng, E.N.; Guo, P.; Guo, L.; Mao, S.S. Surface Engineered Doping of Hematite Nanorod Arrays for Improved Photoelectrochemical Water Splitting. **2014**, 1–9, doi:10.1038/srep06627.

20. Banbela, H.M.; Alharbi, L.M.; Al-Dahiri, R.H.; Jaremko, M.; Salam, M.A. Preparation, Characterization, and Electrochemical Performance of the Hematite/Oxidized Multi-Walled Carbon Nanotubes Nanocomposite. *Molecules* **2022**, *27*, 2708. https://doi.org/10.3390/molecules27092708.

21. Farhanian, D.; Crescenzo, G. De; Tavares, J.R. Large-Scale Encapsulation of Magnetic Iron Oxide Nanoparticles via Syngas Photo-Initiated Chemical Vapor Deposition. *Sci. Rep.* **2018**, 1–11, doi:10.1038/s41598-018-30802-1.

22. Ahmmad, B.; Leonard, K.; Shariful Islam, M.; Kurawaki, J.; Muruganandham, M.; Ohkubo, T.; Kuroda, Y. Green Synthesis of Mesoporous Hematite (α-Fe_2O_3) Nanoparticles and Their Photocatalytic Activity. *Adv. Powder Technol.* **2013**, *24*, 160–167, doi:10.1016/j.apt.2012.04.005.

23. Dolai, J.; Mandal, K.; Jana, N.R. Nanoparticle Size Effects in Biomedical Applications. *ACS Appl. Nano Mater.* 2021, *4*.

24. CLSI *CLSI M100-ED29: 2019 Performance Standards for Antimicrobial Susceptibility Testing, 29th Edition*; 2019;
